# Classifying attentional vulnerability to total sleep deprivation using baseline features of Psychomotor Vigilance Test performance

**DOI:** 10.1038/s41598-019-48280-4

**Published:** 2019-08-20

**Authors:** Eric Chern-Pin Chua, Jason P. Sullivan, Jeanne F. Duffy, Elizabeth B. Klerman, Steven W. Lockley, Bruce S. Kristal, Charles A. Czeisler, Joshua J. Gooley

**Affiliations:** 1 Center for Cognitive Neuroscience, Duke-NUS Medical School, Singapore, 169857 Singapore; 2 Neuroscience and Behavioral Disorders Program, Duke-NUS Medical School, Singapore, 169857 Singapore; 3 Division of Sleep and Circadian Disorders, Departments of Medicine and Neurology, Brigham and Women’s Hospital, Boston, MA 02115 USA; 4 Division of Sleep Medicine, Harvard Medical School, Boston, MA 02115 USA

**Keywords:** Sleep deprivation, Attention

## Abstract

There are strong individual differences in performance during sleep deprivation. We assessed whether baseline features of Psychomotor Vigilance Test (PVT) performance can be used for classifying participants’ relative attentional vulnerability to total sleep deprivation. In a laboratory, healthy adults (n = 160, aged 18–30 years) completed a 10-min PVT every 2 h while being kept awake for ≥24 hours. Participants were categorized as vulnerable (n = 40), intermediate (n = 80), or resilient (n = 40) based on their number of PVT lapses during one night of sleep deprivation. For each baseline PVT (taken 4–14 h after wake-up time), a linear discriminant model with wrapper-based feature selection was used to classify participants’ vulnerability to subsequent sleep deprivation. Across models, classification accuracy was about 70% (range 65–76%) using stratified 5-fold cross validation. The models provided about 78% sensitivity and 86% specificity for classifying resilient participants, and about 70% sensitivity and 89% specificity for classifying vulnerable participants. These results suggest features derived from a single 10-min PVT at baseline can provide substantial, but incomplete information about a person’s relative attentional vulnerability to total sleep deprivation. In the long term, modeling approaches that incorporate baseline performance characteristics can potentially improve personalized predictions of attentional performance when sleep deprivation cannot be avoided.

## Introduction

Many workers experience insufficient sleep due to long and irregular work hours, including military, public safety, and medical personnel^1,2^. Sleep deprivation results in a broad range of performance deficits including decreased vigilance, impaired working memory, and reduced processing speed^3–6^. There are strong trait-like individual differences in responses to sleep deprivation^7,8^. For a given performance task, some individuals consistently show severe cognitive impairment during exposure to sleep loss, whereas others are consistently able to maintain relatively high levels of performance. There is currently no simple approach for estimating how a person’s performance will be affected by sleep deprivation. Development of methodologies for predicting individual responses to sleep deprivation may improve fatigue management and lower risk for occupational errors and accidents in safety-sensitive operational settings.

Several studies have shown that groups of participants categorized as either vulnerable or resilient to sleep deprivation exhibit differences in behavior and/or physiology at baseline (i.e., under rested conditions). Group differences at baseline were observed for functional magnetic resonance imaging (fMRI)-based measures^9–13^, heart rate and its variability^14^, electroencephalogram (EEG) theta activity^14^, and performance on the Psychomotor Vigilance Test (PVT)^14,15^, which is used to assess sustained attention^16^. We and others have shown that a substantial proportion of variance in PVT performance during sleep deprivation can be explained by individual differences at baseline^7,8,17^. In participants who were stratified into high-performing and low-performing groups based on their baseline PVT performance, exposure to total sleep deprivation was associated with a much larger between-group difference in attentional lapses during sleep deprivation compared to when the participants were well rested^14^. These findings suggest that sleep deprivation can amplify individual differences in PVT performance that were already present at baseline. Moreover, these studies suggest that baseline measures of PVT performance can potentially be used to improve predictions of individual differences in attentional responses to sleep deprivation^18^.

The goal of the present study was to develop models that can predict an individual’s relative performance on an attention task to total sleep deprivation assessed under highly controlled laboratory conditions, using features derived from a single 10-min PVT taken at baseline. Performance on the PVT is highly sensitive to sleep deprivation and circadian timing^19–21^, and the number of PVT lapses during sleep deprivation exhibits trait-like stability^7,8^ and substantial broad-sense heritability (h^2^ = 0.83)^22^ when measured in laboratory studies. In addition, the PVT is suitable for repeated administration with few or no learning effects^23^, and can be implemented in diverse operational environments^24–26^. For these reasons, we evaluated whether baseline PVT performance characteristics can be used for predicting a person’s relative vulnerability to worsening attention during a night of sleep deprivation. Using a linear discriminant modeling approach with a wrapper-based method for feature subset selection, we tested the hypothesis that PVT features at baseline (i.e., under rested conditions) can be used to classify participants into performance groups (vulnerable, intermediate, or resilient) that differ in their number of attentional lapses during total sleep deprivation.

## Methods

### Participants and screening procedures

In this retrospective study, we examined data from 185 healthy adults aged 18–30 years who participated in inpatient studies that included an episode of total sleep deprivation (i.e., ≥24 h of continuous wake) in the Intensive Physiologic Monitoring Unit within the Harvard Clinical and Translational Science Center at Brigham and Women’s Hospital (Boston, MA). Data were combined across several previous studies that investigated effects of different types of light stimuli on human circadian rhythms^27–32^. All studies implemented the same screening criteria and laboratory procedures during the sleep deprivation portion of the protocol. Eligibility was assessed by screening questionnaires, a physical examination, blood biochemistry and hematology, an electrocardiogram, and an interview with a clinical psychologist or psychiatrist. Participants who completed the sleep deprivation protocol (n = 185) were considered for inclusion in the present analysis.

Prior to the laboratory study, participants were required to maintain a fixed sleep schedule (8 h of time in bed per night) for at least 1 week. Participants chose a sleep schedule within the range of 2200–0600 (earliest possible schedule) to 0200–1000 (latest possible schedule). Compliance was verified by continuous actigraphy monitoring (Actiwatch-L; Minimitter, Inc., Bend, OR). Participants were ineligible for the laboratory study if their actigraphy-estimated sleep pattern deviated from the prescribed sleep-wake schedule by more than 30 min on any given night. A comprehensive toxicology screen was performed on the day of admission to the laboratory study to ensure that participants had complied with refraining from caffeine, over-the-counter drugs, recreational drugs, alcohol, and nicotine use. Female participants underwent a blood test to verify that they were not pregnant. Informed written consent was obtained from all participants, and research procedures were approved by the Human Research Committee at Partners HealthCare and complied with HIPAA regulations and ethical guidelines in the Declaration of Helsinki.

### Protocol overview

Research participants were studied individually in a laboratory environment that was free of time cues. During the first 3 days of the laboratory study, participants were scheduled to sleep and wake at their pre-study sleep-wake times. Participants slept in darkness and were exposed to <200 lux during wake episodes until midway through the third day, after which time the lights were dimmed to <3 lux measured at eye level. Ambient light was provided by ceiling mounted 4100 K lamps (Philips Lighting, Eindhoven, The Netherlands) with illuminance measured using a portable radiometer (International Light Technologies ILT1400) as previously described^27,28^. Beginning on the fourth morning of the study, participants were kept awake continuously for 30–50 h using constant routine procedures^33^. During this episode of total sleep deprivation, wakefulness was enforced by a staff member who was present in the participant’s room. The staff member also monitored the participant’s compliance with research procedures, including computer testing. Participants were required to maintain a semi-recumbent position in bed under dim ambient lighting (<3 lux), with small equicaloric snacks given every hour. Following exposure to total sleep deprivation, participants had a recovery sleep episode and an additional 4–5 days of their study (results reported elsewhere)^27–31^.

### Psychomotor vigilance test

Participants completed a 10-min Psychomotor Vigilance Test (PVT) every 2 h while awake, including the first 3 days of the study and throughout the sleep deprivation protocol. The PVT is a simple reaction time test that is used to assess sustained visual attention^16,23^. Participants were instructed to maintain their fastest possible reaction time to a visual stimulus that was presented on a computer monitor at random inter-stimulus intervals ranging from 1–9 s. The stimulus comprised a count-up timer that counted up in milliseconds until the participant pressed a button on a dedicated PVT response box with the thumb of their dominant hand. Attentional lapses were defined as reaction times that exceeded 500 milliseconds^6,8,34,35^.

Of the 185 participants whose data were considered for the present study, 25 participants were excluded from our analyses due to missing or invalid PVT data during the episode of total sleep deprivation (from 16 to 24 h after wake-up time). Participants were excluded from retrospective data selection if they had one or more PVT sessions that were missing or invalid for the 5 sessions that took place during sleep deprivation (i.e., 16, 18, 20, 22, or 24 h after wake-up time) because these data were used to categorize participants into different vulnerability groups (see below). Missing data were defined as PVT sessions that did not take place or were not digitally recorded due to procedural or technical problems. Invalid PVT sessions were defined as those in which the PVT was administered more than an hour later than the scheduled time (e.g., due to procedural or technical problems), the participant used the incorrect button on his/her PVT response box for >10% of trials for a given PVT session, no PVT reaction time was recorded for >1 min during a given PVT session (e.g., due to the participant not responding or a technical problem), or the 10-min PVT session was truncated to <8 min due to a technical problem (e.g., if the computer program crashed during a PVT session). Using these pre-determined criteria for retrospective data selection, there were 8 participants who did not complete all of the PVTs at the scheduled times due to procedural problems; 8 participants who were excluded due to experimental problems that invalidated their data; and 9 participants who were excluded due to incomplete PVT data. Hence, 160 participants were included in the present study (65% male; mean age ± SD = 23.0 ± 2.8 years).

### Model for classifying vulnerability to total sleep deprivation

#### Stratification of participants into different vulnerability groups

To categorize participants as resilient or vulnerable to total sleep deprivation, we ranked them by their performance when they were sleep deprived, as indexed by their average number of PVT lapses (reaction times > 500 ms) during hours 16 to 24 of the sleep deprivation constant routine procedure. The top quartile of participants with the fewest number of lapses was defined as the resilient group (n = 40) and the bottom quartile with the greatest number of lapses was defined as the vulnerable group (n = 40). The middle 50% of participants whose performance fell within the interquartile range were defined as the intermediate performance group (n = 80).

#### Identification of candidate baseline PVT features

Baseline performance on the PVT was assessed during the first 16 hours of wakefulness of the constant routine procedure. There were 6 PVT sessions during the baseline interval (4 h, 6 h, 8 h, 10 h, 12 h, and 14 h after wake-up time). For each PVT session that a participant completed, 426 summary measures were calculated based on trial-by-trial reaction times. These measures characterized various aspects of PVT performance, including speed and variability of reaction times, response errors (e.g., anticipation errors or failure to respond), and time-on-task effects (Table S1). In addition to standard PVT performance metrics^36^, we included measures based on those introduced recently by our group and others, including percentile reaction times^37^ and lapse counts based on reaction time thresholds that reflect different probabilities of the participant’s eyes being closed when lapses occurred^38^. We also included a series of novel non-parametric measures of reaction time variability, such as the number of times that the difference of consecutive reaction times exceeded a particular threshold, and differences of percentile reaction times.

Baseline PVT features that are consistent over repeated assessments (within and between participants) are more likely to be reliable in predicting responses during sleep deprivation compared to features that are more variable. We therefore pre-selected candidate baseline PVT features for our models that exhibited stable individual differences across multiple days of rested wakefulness (day 2, day 3, and the first 16 h of wakefulness on day 4). PVT measures were chosen as candidate features for the linear discriminant model (see below) if they met the pre-determined criterion of having an intra-class correlation coefficient (ICC) value > 0.60, which corresponds to the threshold for ‘substantial’ or ‘good’ agreement between measurements^39,40^. For each PVT measure at each time point, the ICC value was calculated as between-participant variance (σ^2^_BS_) divided by the sum of between- and within-participant variance (σ^2^_BS_ + σ^2^_WS_), with values ranging from 0 to 1. An ICC value was computed separately for each baseline PVT measure at each time point (4 h, 6 h, 8 h, 10 h, 12 h, and 14 h after wake-up time). A linear mixed model was used with day (day 2, 3, and 4) as a fixed factor to correct for systematic order effects (i.e., to account for day-to-day changes in PVT performance that might be related to the research protocol), and between-participant differences were modeled as a random intercept with a Gaussian distribution^41^. Across baseline PVT sessions, about one third of PVT measures met the criterion of having an ICC value > 0.60 and were considered for feature subset selection (number of PVT measures with an ICC value > 0.60: 4 h, 165 measures; 6 h, 108 measures; 8 h, 134 measures; 10 h, 135 measures; 12 h, 163 measures; 14 h, 137 measures). Metrics with an ICC value < 0.60 were excluded and were not revisited in the current study.

#### Three-class linear discriminant model

For each baseline time point (4 h, 6 h, 8 h, 10 h, 12 h, and 14 h after wake-up time on day 4 of the protocol), a different linear discriminant classification model was developed to predict individuals in resilient, intermediate, and vulnerable performance groups. A wrapper-based method was used to determine the optimal feature subset for predicting performance during total sleep deprivation^42^. In short, a wrapper approach assesses the relative usefulness of candidate feature subsets through its estimated prediction performance, and incorporates downstream prediction performance to obtain an optimized feature subset. We used a forward selection, best-first search algorithm to perform the feature set search^42,43^.

Within the wrapper best-first selection, for a given candidate feature set we added an additional layer to evaluate model accuracy using cost matrices from 1 to 5 (in 0.5 steps)^44^. A cost matrix assigns weights to different outcomes, hence penalizing and protecting against specific types of misclassification errors. In the present study, we did not commit to a particular cost matrix because the choice is user-context dependent. For example, in some operational settings it may be more costly (i.e., undesirable) to misclassify a person who is vulnerable to sleep deprivation as resilient, as compared with misclassifying a person who is resilient as vulnerable. Our modeling approach searched for the optimal combination of feature set and cost matrix to optimize prediction accuracy, which was used as the performance metric to select the model for the three-class prediction.

For a given trial feature set, we used repeated 5-fold cross validation to evaluate its performance. Fold assignment was performed for the resilient, intermediate, and vulnerable groups separately, i.e., stratified, to ensure the proportion of participants in each group stayed constant across folds. Prediction performance across 100 repeated cross-validation runs was averaged to reduce the variance expected of 5-fold cross-validation. The best-first search algorithm was terminated upon reaching the condition in which performance did not improve for 5 consecutive iterations. After the best feature subset and cost matrix was determined for a given PVT session, the final classification model was determined by applying the best feature subset on the entire initial dataset.

For each PVT session completed at baseline (4 h, 6 h, 8 h, 10 h, 12 h, and 14 h after wake-up time), its corresponding model arranged the summary PVT data (which initially consisted of data expressed in different types of units) as a row vector **d**, and normalized the data for a given PVT measure across subjects by z-scoring them, i.e. **z** = (**d** − **μ**)/**σ**, where **μ** and **σ** are the z-score parameters. Then, for each of the possible outcomes, i.e., resilient, intermediate, or vulnerable, the model computed the posterior probability that the participant belonged to that group, given the set of model parameters and cost matrix (Table S2). Specifically, the discriminant functions for each group, i.e., the natural logarithm of the posterior probabilities, were computed as linear combinations of the summary PVT measures, according to:

$$\begin{array}{rcl}{{\rm{g}}}_{{\rm{R}}} & = & {{\bf{w}}}_{{\rm{R}}}{{\bf{z}}}^{{\rm{T}}}+{{\rm{c}}}_{{\rm{R}}}\\ {{\rm{g}}}_{{\rm{I}}} & = & {{\bf{w}}}_{{\rm{I}}}{{\bf{z}}}^{{\rm{T}}}+{{\rm{c}}}_{{\rm{I}}}\\ {{\rm{g}}}_{{\rm{V}}} & = & {{\bf{w}}}_{{\rm{V}}}{{\bf{z}}}^{{\rm{T}}}+{{\rm{c}}}_{{\rm{V}}}\end{array}$$

In each equation, **g** is the discriminant function, **w** is the coefficient, and **c** is the constant term (R = resilient; I = intermediate; V = vulnerable). Participants were assigned to the group with the highest value for the discriminant function, which is equivalent to assignment based on the highest posterior probability. Depending on the testing time point, a different set of z-score and model parameters was applied.

### Assessment of model performance

Performance of the models was assessed using several metrics: (1) Accuracy of the classifier, defined as the proportion of participants who were correctly classified as resilient, intermediate, or vulnerable; (2) Cohen’s kappa, which measures accuracy after discounting agreement by chance, i.e., accounting for the 1:2:1 structure of the outcome assignments (chance-level accuracy was 37.4% for the 3-class classification task); (3) Sensitivity, defined as the proportion of vulnerable participants who were correctly classified as vulnerable (for the vulnerable classifier), or as the proportion of resilient participants who were correctly classified as resilient (for the resilient classifier); (4) Specificity, defined as the proportion of intermediate and resilient participants who were correctly identified as not being vulnerable (for the vulnerable classifier), or as the proportion of intermediate and vulnerable participants who were correctly identified as not being resilient (for the resilient classifier); (5) Positive predictive value, defined as the proportion of participants with a test outcome (i.e., predicted outcome) of vulnerable who were truly vulnerable (for the vulnerable classifier), or as the proportion of participants with a test outcome of resilient who were truly resilient (for the resilient classifier); and (6) Negative predictive value, defined as the proportion of participants with a test outcome of either resilient or intermediate who were truly resilient or intermediate (for the vulnerable classifier), or as the proportion of participants with a test outcome of either intermediate or vulnerable who were truly intermediate or vulnerable (for the resilient classifier). Analyses and model development were implemented by self-written code using MATLAB software (MATLAB 2012a; MathWorks, Inc, Natick, MA).

## Results

### Characterization of vulnerability to total sleep deprivation

**Figure 1:**
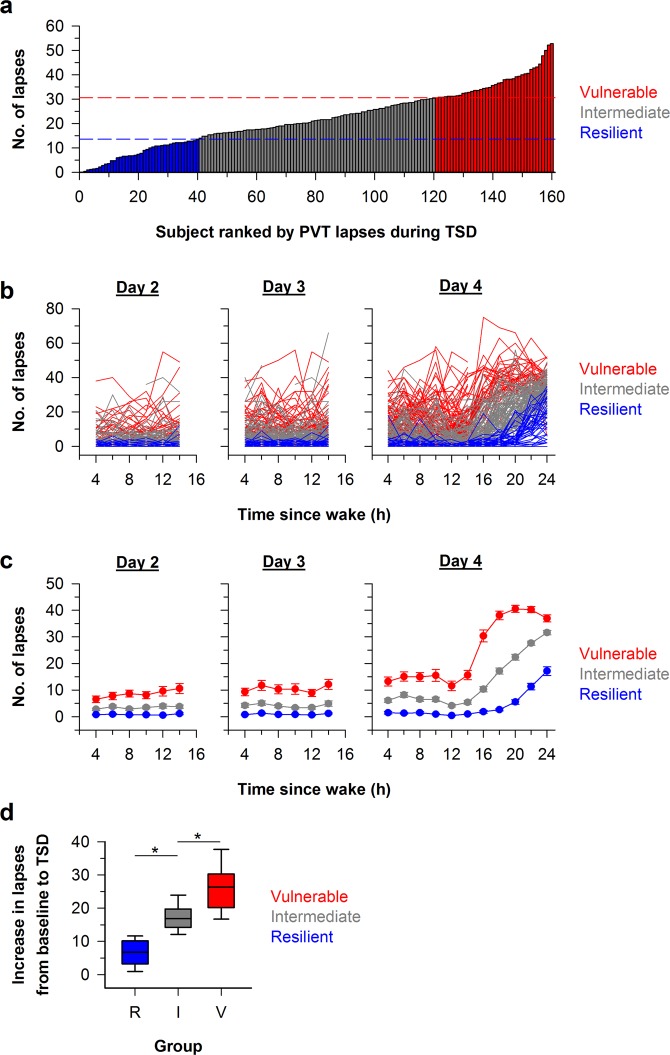
Stratification of participants into resilient, intermediate, and vulnerable performance groups. (**a**) The average number of lapses (reaction times > 500 ms) on the Psychomotor Vigilance Test (PVT) during total sleep deprivation (TSD; 16 h to 24 h after wake-up time) is shown for 160 participants who were categorized into vulnerable (red, n = 40), intermediate (gray, n = 80), and resilient (blue, n = 40) performance groups. Dashed lines demarcate thresholds (25^th^ percentile and 75^th^ percentile) used to define vulnerable and resilient groups. (**b**) The time course of PVT lapses is shown on baseline days that preceded TSD (Day 2 and Day 3) and during 24 h of continuous wakefulness (Day 4). (**c**) The mean ± SEM is shown for PVT lapses in groups of participants categorized as vulnerable, intermediate, or resilient during TSD. (**d**) Box plots show the increase in the number of lapses from baseline to sleep deprivation in participants whose performance was categorized as Resilient (R), Intermediate (I), or Vulnerable (V) during TSD. Boxes show the median and interquartile range, and whiskers indicate the 95^th^ percentile of the distribution. Asterisks indicate significant between-group differences (P < 0.001).

In our sample of 160 young healthy adults, large individual differences in PVT performance were observed during exposure to total sleep deprivation (Fig. 1a). The quartile of participants who were defined as vulnerable had an average of 30.6 to 52.8 lapses per PVT session; the quartile of participants who were defined as resilient had an average of 0.0 to 13.6 lapses per PVT session; and the remaining group of participants with intermediate performance had an average of 14.0 to 30.4 lapses per PVT session. Based on our stratification scheme for defining performance during total sleep deprivation, there was almost no overlap in PVT lapses across individual sessions for participants who were categorized as vulnerable versus resilient (Fig. 1b).

There was a significant difference between groups in the increase in PVT lapses from baseline to sleep deprivation (Fig. 1c,d; Kruskal-Wallis Test, H = 106.0, P < 0.001). Specifically, the group that was categorized as vulnerable based on lapses during total sleep deprivation exhibited a greater increase in PVT lapses from baseline compared with the intermediate performance group, despite having a greater number of lapses at baseline (Dunn’s post test, Q = 4.7, P < 0.05). Hence, the group we defined as vulnerable was more susceptible to sleep deprivation, even after taking into account baseline differences in PVT performance. Similarly, the intermediate performance group exhibited a greater increase in lapses compared with the resilient group (Dunn’s post test, Q = 7.0, P < 0.05). These results show that our definition for performance vulnerability, which was based on the average number of lapses during total sleep deprivation, also reflects differences in the magnitude of deterioration in performance from baseline to the sleep-deprived state.

### Model for predicting performance vulnerability using baseline PVT measures

Separate linear discriminant models were developed using baseline PVT features from 6 different time points (4 h, 6 h, 8 h, 10 h, 12 h, and 14 h after wake-up time; Table S2) to classify participants in resilient, intermediate, and vulnerable performance groups. Across the different models, classification accuracy ranged from 65% to 75%, and Cohen’s kappa ranged from 0.48 to 0.61 (Table 1), indicating ‘moderate’ strength of agreement^40^ between the classification model and the true status of participants (i.e., vulnerable, intermediate, resilient). Then, we assessed how well each model performed at the binary classification task of classifying resilient participants (top quartile) versus the bottom 75% of performers during total sleep deprivation (Table 1). Models that were developed across the 6 baseline PVT sessions provided about 78% sensitivity (range 63–90%), 86% specificity (range 75–93%), 68% positive predictive value (range 55–78%), and 93% negative predictive value (range 89–96%). We also assessed the performance of each model at classifying vulnerable participants (bottom quartile) versus the top 75% of performers. For this classification task, the models provided about 70% sensitivity (range 57–84%), 89% specificity (range 83–94%), 70% positive predictive value (range 60–79%), and 90% negative predictive value (range 87–94%).

**Table 1:** Model performance metrics in the full sample (n = 160).

Metric	Time since wake					
	4 h	6 h	8 h	10 h	12 h	14 h
Accuracy (%)	70.4 ± 1.7	66.7 ± 1.7	75.6 ± 1.9	72.3 ± 1.6	69.9 ± 2.1	64.8 ± 1.4
Cohen’s kappa	0.51 ± 0.03	0.50 ± 0.03	0.61 ± 0.03	0.54 ± 0.03	0.55 ± 0.03	0.48 ± 0.02
***Classifying resilient participants versus bottom 75% of performers***						
Sensitivity (%)	62.9 ± 3.5	84.0 ± 3.0	81.0 ± 2.7	69.2 ± 2.4	83.7 ± 4.1	89.7 ± 2.7
Specificity (%)	91.0 ± 1.3	84.9 ± 1.2	90.7 ± 0.92	92.8 ± 1.2	81.4 ± 1.6	75.1 ± 1.2
Positive predictive value (%)	71.8 ± 3.6	66.9 ± 2.4	76.0 ± 2.2	77.6 ± 2.9	60.7 ± 2.7	55.0 ± 1.9
Negative predictive value (%)	88.5 ± 1.0	94.2 ± 1.0	93.7 ± 0.83	90.3 ± 0.76	94.1 ± 1.4	95.9 ± 1.0
***Classifying vulnerable participants versus top 75% of performers***						
Sensitivity (%)	57.0 ± 3.4	73.9 ± 2.4	70.0 ± 3.5	57.4 ± 3.3	84.0 ± 4.2	79.6 ± 2.1
Specificity (%)	91.8 ± 1.2	83.2 ± 1.4	91.2 ± 1.7	94.4 ± 1.2	86.7 ± 1.5	85.4 ± 1.2
Positive predictive value (%)	71.4 ± 3.7	60.3 ± 2.6	74.6 ± 4.1	79.1 ± 5.0	69.3 ± 3.3	66.0 ± 2.5
Negative predictive value (%)	86.9 ± 0.94	90.9 ± 0.85	90.3 ± 1.1	87.2 ± 0.86	94.4 ± 1.4	92.8 ± 0.74

A 3-class linear discriminant model was developed using features of Psychomotor Vigilance Test performance at each baseline time point to classify participants in different vulnerability groups (vulnerable, intermediate, resilient). For each model performance metric, the mean ± SD is shown for 100 runs of stratified 5-fold cross validation.

**Figure 2:**
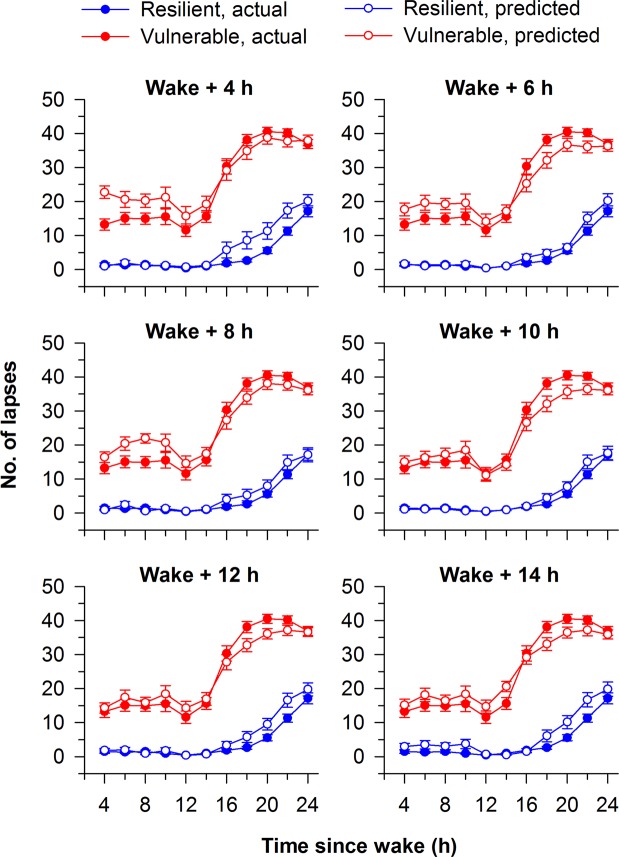
PVT lapses (reaction times > 500 ms) in groups of participants predicted to be resilient or vulnerable to total sleep deprivation. Models were developed using baseline PVT performance features to classify individuals as resilient (blue, open circles) or vulnerable (red, open circles) to the effects of total sleep deprivation. Separate models were developed using a 10-min PVT performed at 6 different time points (4 h, 6 h, 8 h, 10 h, 12 h, and 14 h after habitual wake time). In all models and at every time point, P < 0.001 for pairwise comparisons between predicted resilient and vulnerable groups. For comparison, the time course of PVT lapses is shown for participants categorized as resilient or vulnerable based on their average number of lapses during total sleep deprivation (resilient, blue circles; vulnerable, red circles). The mean ± SEM is shown in each panel.

Next, we compared the time course of PVT lapses in groups of participants classified as either vulnerable or resilient to the effects of total sleep deprivation. The goal of this analysis was to assess qualitatively the ability of the models to select for groups of participants enriched for either vulnerability or resilience. For the classification model developed at each baseline time point, we performed 100 runs of 5-fold cross validation, resulting in 100 predictions for a given participant. The most common prediction (i.e., the mode) in each individual was used to determine his/her group assignment (vulnerable, intermediate, or resilient). For classification models developed at each of the 6 baseline PVT sessions, the time course of PVT lapses was markedly different between the predicted vulnerable and resilient groups (Fig. 2; For comparisons between groups at every time point and model, P < 0.001), indicating that baseline features of PVT performance could be used to discriminate groups of participants who differed in their attentional vulnerability during total sleep deprivation.

### Predicting performance vulnerability in participants with high performance at baseline

Our modeling approach was based on the premise that baseline individual differences in performance carry information about attentional responses during total sleep deprivation. Given that there were substantial individual differences in PVT performance at baseline in the full sample (n = 160), we sought to establish whether our modeling approach could be used to predict vulnerability to total sleep deprivation in a group of participants with less heterogeneity in PVT performance (i.e., high-performing subjects). We therefore performed a post-hoc stratification and analysis in which we applied our modeling approach to the subset of participants who exhibited ≤ 2 lapses per PVT session at baseline (n = 60). In this subgroup of high-performing individuals, we stratified participants according to the same criteria as those used in the full sample, i.e. the quartile of participants with the greatest number of PVT lapses during sleep deprivation was defined as vulnerable (n = 15), and the quartile with the fewest number of lapses was defined as resilient (n = 15).

Using the same modeling approach described for the full dataset, we developed a set of new models in the subgroup with ≤ 2 lapses at baseline (4 h, 6 h, 8 h, 10 h, 12 h, and 14 h after wake-up time). In these models, classification accuracy ranged from 57% to 64%, and Cohen’s kappa ranged from 0.38 to 0.46, indicating ‘fair-to-moderate’ strength of agreement^40^ between the classification model and participants’ actual vulnerability status (Table 2). For classification of resilient participants (top quartile) versus the bottom 75% of performers during sleep deprivation, our models provided about 69% sensitivity (range 49–90%), 80% specificity (range 75–89%), 56% positive predictive value (range 45–76%), and 90% negative predictive value (range 83–97%). For classification of vulnerable participants (bottom quartile) versus the top 75% of performers, the models provided about 80% sensitivity (range 67–87%), 76% specificity (range 68–81%), 56% positive predictive value (range 47–62%), and 93% negative predictive value (range 89–95%).

**Table 2:** Model performance metrics in participants with ≤ 2 lapses at baseline (n = 60).

Metric	Time since wake					
	4 h	6 h	8 h	10 h	12 h	14 h
Accuracy (%)	63.2 ± 3.3	57.2 ± 1.6	59.8 ± 3.5	59.1 ± 3.9	64.3 ± 3.5	61.3 ± 3.1
Cohen’s kappa	0.46 ± 0.05	0.38 ± 0.02	0.39 ± 0.05	0.39 ± 0.06	0.46 ± 0.06	0.44 ± 0.05
***Classifying resilient participants versus bottom 75% of performers***						
Sensitivity (%)	90.1 ± 4.1	60.3 ± 1.6	48.5 ± 4.8	61.4 ± 6.2	80.1 ± 8.4	74.7 ± 3.8
Specificity (%)	88.6 ± 3.3	74.7 ± 1.4	78.4 ± 3.6	75.5 ± 3.9	83.7 ± 1.5	75.8 ± 3.2
Positive predictive value (%)	76.3 ± 5.5	46.5 ± 4.4	45.3 ± 7.9	47.5 ± 7.2	65.9 ± 5.3	53.5 ± 5.4
Negative predictive value (%)	97.1 ± 1.1	85.6 ± 1.1	82.5 ± 1.8	86.5 ± 2.3	93.2 ± 2.8	90.8 ± 1.5
***Classifying vulnerable participants versus top 75% of performers***						
Sensitivity (%)	78.7 ± 5.0	86.7 ± 0.0	83.1 ± 4.5	80.1 ± 7.9	67.1 ± 5.8	83.3 ± 8.7
Specificity (%)	67.5 ± 4.2	72.5 ± 1.6	80.9 ± 1.7	79.4 ± 2.5	79.7 ± 3.8	76.4 ± 1.6
Positive predictive value (%)	47.0 ± 4.8	54.6 ± 3.1	62.2 ± 4.2	58.9 ± 5.5	56.4 ± 6.8	57.3 ± 5.0
Negative predictive value (%)	91.3 ± 2.2	94.8 ± 0.66	94.1 ± 1.5	93.0 ± 2.8	88.5 ± 2.1	94.1 ± 3.0

A 3-class linear discriminant model was developed to classify participants in different vulnerability groups (vulnerable, intermediate, resilient) using features of baseline Psychomotor Vigilance Test (PVT) performance. A separate model was developed for each PVT taken during the baseline period and was applied only to high-performing participants with ≤2 lapses at baseline. For each model performance metric, the mean ± SD is shown for 100 runs of stratified 5-fold cross validation.

**Figure 3:**
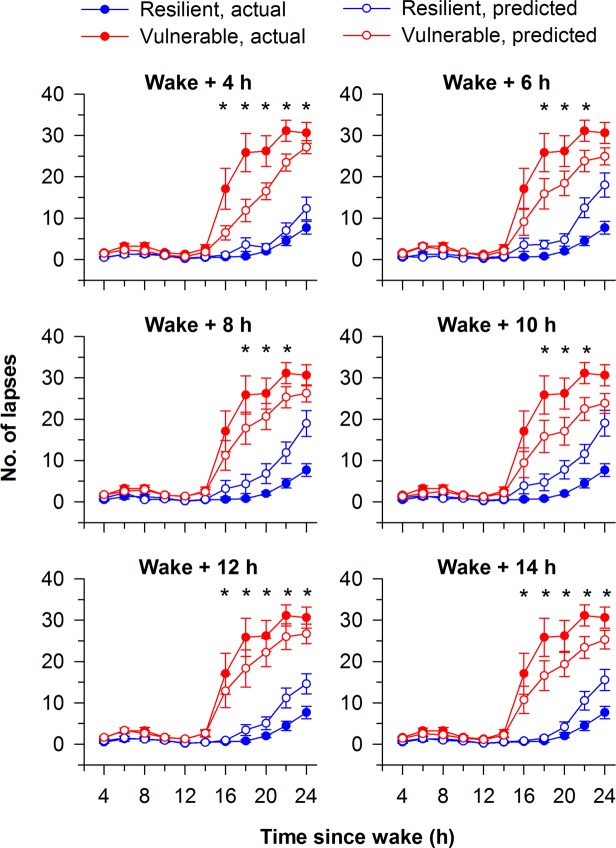
PVT lapses (reaction times > 500 ms) in groups of participants with high performance at baseline (≤2 lapses; n = 60) who were classified as resilient or vulnerable to total sleep deprivation. Participants were classified as resilient (blue, open circles) or vulnerable (red, open circles) to the effects of total sleep deprivation using models developed on baseline PVT measures. Separate models were developed using a 10-min PVT performed at 6 different time points (4 h, 6 h, 8 h, 10 h, 12 h, and 14 h after habitual wake time). Asterisks (*) indicate significant pairwise differences in PVT lapses between predicted resilient and vulnerable groups during the sleep deprivation period (P < 0.05). For comparison, the time course of PVT lapses is shown for participants categorized as resilient or vulnerable based on their average number of lapses during total sleep deprivation (resilient, blue circles; vulnerable, red circles). The mean ± SEM is shown in each panel.

In the subgroup of participants with ≤2 lapses at baseline, we evaluated the average time course of PVT lapses in groups of participants who were classified as either resilient or vulnerable to total sleep deprivation. This was assessed for models developed for each baseline time point. Similar to results in the full sample, these models were able to classify participants who were high performers at baseline into groups that differed in their attentional responses to total sleep deprivation (Fig. 3). Irrespective of the time point of PVT testing that was used to develop each model, the group of participants that was predicted to be more vulnerable to sleep deprivation exhibited more lapses than the group that was predicted to be resilient to the effects of sleep deprivation.

## Discussion

In the present study, we developed and tested a series of models for predicting individual differences in attentional vulnerability to total sleep deprivation. Using features derived from a single 10-min PVT taken under rested conditions, participants in the full dataset (n = 160) were classified in vulnerable, intermediate, and resilient performance groups with about 70% accuracy across models developed at different baseline time points (range, 65–76%). These results demonstrate that baseline individual differences in PVT performance metrics associate with relative vulnerability to total sleep deprivation. The robustness of our modeling approach was assessed in a subgroup of high-performing persons (≤2 lapses at baseline), in whom baseline individual differences were much smaller compared with the full sample. As expected, performance of the classifier dropped in this subgroup, but the model was still able to classify participants in different performance groups (vulnerable, intermediate, or resilient) with about 60% accuracy for models developed across different baseline PVT sessions (range, 57–64%), i.e., above a chance-level prediction (37.4%). Hence, even in top-performing participants, individual differences in baseline performance associated with responses to total sleep deprivation.

In our prior work based on data collected from a different group of participants^14^, we showed that individuals who were categorized as vulnerable to the effects of total sleep deprivation on PVT lapses exhibited slower and more variable reaction times at baseline. This led us to hypothesize that exposure to sleep deprivation may contribute to enhancement of individual differences in PVT performance that were already present at baseline^14^. In a follow-up analysis of participants in that study who were exposed to total sleep deprivation on 2 separate occasions, we showed that individual differences in baseline PVT performance contributed significantly to between-participant variance in performance during sleep deprivation^17^. Based on variance components analysis of PVT performance, nearly 50% of the variance in performance during exposure to total sleep deprivation was explained by individual differences at baseline. Consequently, the intra-class correlation coefficient value dropped substantially when adjusting for baseline performance as a covariate. Although baseline individual differences did not fully account for individual differences in PVT performance during prolonged wakefulness^7,8^, we hypothesized that baseline differences in PVT performance could nonetheless be used to estimate a person’s relative vulnerability to total sleep deprivation. The present study addressed this question, demonstrating that various measures of baseline PVT performance could be used to classify participants’ vulnerability to total sleep deprivation with nearly double the accuracy compared with chance-level prediction accuracy (i.e., much greater than 37.4% chance-level prediction for the 3-class classification task).

Most predictive performance models that have been developed have been used to identify critical times of performance vulnerability in an average individual (i.e., based on group data)^45–48^, rather than providing individualized predictions of performance under conditions of sleep loss or circadian misalignment. Adaptive individual-specific performance models address this issue^49–52^, but such models rely on repeated assessments of PVT performance over time under sleep loss conditions. They approach the problem by assessing a person’s responses to sleep deprivation periodically and updating the model to predict his/her responses to subsequent sleep deprivation, rendering it an impractical approach for many situations. More recently, drift diffusion model^53^ parameters derived from baseline PVT data have been used to classify participants in resilient and vulnerable groups with 77% accuracy using a support vector machine (SVM) modeling approach^18^. In its current form, however, that model requires data to be combined across consecutive PVTs for reliable estimates of drift diffusion parameters, whereas our classifier relies on data derived from a single 10-min PVT. Additionally, participants in that study were categorized as vulnerable or resilient to sleep deprivation based on a median split of PVT lapses after exposure to sleep deprivation. Using such a definition, there is little separation between higher-performing vulnerable participants and lower-performing resilient participants. By comparison, our modeling approach included an intermediate performance group, hence ensuring that participants who were categorized as vulnerable or resilient to the effects of total sleep deprivation on PVT performance were phenotypically distinct. The slightly lower classification accuracy in our study might be explained in part by the more challenging 3-class classification task (37.4% versus 50.0% chance-level prediction) and the use of a single PVT session for building the classifier.

The performance and generalizability of our modeling approach are likely influenced by the definitions used for vulnerability to sleep deprivation. PVT lapses have been widely used in sleep and circadian rhythms research to assess sustained attention performance and to model group-level or individual responses to different types of sleep deprivation^15,18,49–52,54,55^. There are, however, other PVT-based metrics that have been proposed for quantifying performance impairment that may better capture the influence of homeostatic and circadian processes^56^. Recent work has shown that PVT metrics derived from diffusion modeling (e.g., the log transformation of the signal-to-noise ratio) have better psychometric properties than PVT lapses, including higher sensitivity, stability, degree of normality, and absence of floor and ceiling effects^57^. Hence, future modeling efforts should consider including other metrics of PVT performance for measuring and predicting changes in vigilant attention.

Consistent with previous studies, we observed strong individual differences in PVT performance during total sleep deprivation^2,8^. Prior work has demonstrated that these individual differences are partially explained by genetic make-up^22,58^, but are likely modulated by other physiologic and behavioral factors. The earlier deterioration in PVT performance during sleep deprivation in participants categorized as vulnerable could be explained by a faster buildup of homeostatic sleep pressure, or an earlier circadian increase in sleep propensity. The present study did not, however, examine effects of individual differences in sleep pressure, circadian phase, or chronotype on the time-course of PVT performance. We also cannot determine the degree to which individual differences in performance were affected by participants’ motivation or effort to perform their best. Effort allocation on the PVT is sensitive to sleep deprivation and reward^59,60^, and is also likely influenced by an individual’s level of intrinsic motivation.

We must also emphasize that individual differences in PVT lapses do not necessarily correspond with individual differences in performance on other cognitive tasks, e.g. those that assess working memory or processing speed^8,61^. As such, our modeling approach may only be relevant for estimating performance deterioration on tasks in which sustained attention is the most important determinant of task performance. It is also well-established that performance on the PVT does not associate with individual differences in self-reported sleepiness^8,14,62^, indicating a dissociation between how sleepy participants feel and how well they can perform during exposure to sleep deprivation. Nonetheless, it has been shown that PVT lapses correlate with physiologic indicators of sleepiness, including percentage eyelid closure over the pupil over time (PERCLOS), low-frequency EEG activity, and some measures of heart rate variability^14^. Thus, PVT performance reflects overall vigilance, with longer reaction times and lapses associated with increased drowsiness and intermittent intrusion of sleep^23,38^.

The performance of our models may be influenced by our definition of baseline PVT performance. During the pre-study screening process, we attempted to minimize the possibility that participants were sleep-deficient before enrolling in the laboratory study. Specifically, participants were instructed to spend 8 h of time in bed for sleep each night for at least one week, and the same sleep schedule was enforced during the first 3 nights of the laboratory study. This amount of time in bed for sleep falls in the middle of the range recommended by the National Sleep Foundation for young adults (7–9 h)^63^, but the duration of sleep required for optimal performance likely varies across individuals. In our previous work we showed that participants who were categorized as vulnerable to the effects of total sleep deprivation on PVT performance exhibited longer sleep durations and more regular sleep patterns under free-living conditions, as compared with participants categorized as resilient^14^. Therefore, we cannot exclude the possibility that participants in the current study who were categorized as vulnerable to total sleep deprivation needed more than 8 h of time in bed for sleep each night for optimal performance^54,64^, and were getting insufficient sleep during the 10+ days immediately prior to undergoing sleep deprivation in the laboratory. Relatedly, the duration of time participants maintained their fixed pre-study sleep-wake schedule ranged from 1 to 3 weeks and was not factored into our analyses. Additionally, the fixed sleep-wake schedule that participants chose did not necessarily reflect their preferred sleep timing and may have been influenced by factors such as work hours, school schedule (for participants who were college students), and family responsibilities and/or home environment. It is also possible that baseline PVT performance was influenced by carry-over effects from sleep history before screening. In future studies, these issues can potentially be addressed by having participants undergo a sleep-extension protocol in which they are given extended opportunities for sleep to minimize any sleep debt^20^, prior to being exposed to sleep deprivation.

There are several sources of selection bias in our study related to participant recruitment. Our participants were selected to be young, very healthy, and willing to undergo rigorous screening and laboratory procedures. The latter included staying in a highly-controlled laboratory setting for 9–10 days, during which participants were exposed to total sleep deprivation, frequent computer testing, and frequent intravenous blood draws (data reported elsewhere) without access to caffeine. Hence, our findings are based on a non-random sample of individuals who may differ in health, personality, lifestyle, and socioeconomic status from the general population. Our participant selection and laboratory procedures likely served to minimize sources of variance in PVT performance, and hence the model performance metrics in the present study may reflect the best-case scenario for predicting individual differences in attentional lapses during total sleep deprivation. Looking forward, it will be important to test our modeling approach prospectively in more diverse groups of participants who differ in age, health, caffeine intake, and sleep history.

Our analyses were limited to a single overnight period of exposure to total sleep deprivation. While pulling an all-nighter is common among students and shift workers, longer episodes of sleep deprivation can occur, e.g. in medical and military personnel. Future modeling efforts should therefore consider both overnight and extended periods of sleep deprivation. While it has been demonstrated that individual differences in PVT performance are preserved across different types of sleep deprivation (total sleep deprivation versus chronic sleep restriction)^7^, it will also be important to test whether our modeling approach can be used to classify performance vulnerability in individuals exposed to chronic sleep restriction and/or irregular sleep schedules^26,55^.

The present study used a linear discriminant model that incorporated a best-first search algorithm with wrapper-based heuristics, which represents an iterative fitting and testing process. An advantage of wrapper methods for feature selection is that they can provide for high classification accuracy^42^. Such methods can, however, result in overfitting if the training data are modeled too well (i.e., modeling peculiarities in the data) resulting in a feature set with poor prediction performance in other datasets. Given that we used an internal cross-validation scheme and did not test the generalizability of our modeling approach using data derived from other experiments or in a hold-out set, our results for accuracy are likely optimistically biased, representing the upper limit of prediction performance that can be achieved. This limitation implies that further improvement will require the discovery and implementation of new metrics (e.g., from physiologic data). There are many other modeling approaches that can be used to select predictor variables and classify participant performance. A comparison of our model with other classifiers was beyond the scope of the present study, but prior attempts to classify vulnerability to sleep deprivation based on PVT performance have included SVM models^18^ and pattern classification based on a k-nearest neighbor algorithm and a Naïve Bayes classifier^55^. Additionally, there are alternative approaches for feature selection that may perform better at finding all relevant features, while discarding those that are redundant, e.g. the Boruta algorithm which uses a wrapper approach built around a random forest classifier^65^. While our approach was successful in discriminating groups of individuals who differed in their attentional vulnerability to total sleep deprivation, future work should investigate the performance of alternative models that may be more robust (e.g., more generalizable and resistant to noise) and amenable to protection against over-fitting. It is also possible that combining outputs from multiple models (e.g., model fusion or ensemble classifiers) may improve classification performance^66^.

In the present study, it is likely that some PVT measures included in each candidate feature set carried redundant or overlapping information. It is therefore unsurprising that related but non-identical PVT features were selected by models developed at each time point (4 h, 6 h, 8 h, 10 h, 12 h, and 14 h after wake-up time). In all models, PVT features were selected that measured the difference in reaction times across different percentiles of the reaction time distribution. In particular, differences in reaction times from the faster-to-middle part of the distribution (e.g., difference in 20^th^ to 55^th^ percentile reaction times) were selected most often across models, with a narrower distribution of reaction times associated with greater resilience to the effects of total sleep deprivation. Measures of reaction time variability (e.g., standard deviation of reciprocal reaction time) and differences in reaction times between fast and slow tails of the distribution (e.g., difference in 15^th^ to 85^th^ percentile reaction times) were also selected in some models, suggesting that distribution-based measures of PVT performance may be especially useful for estimating individual responses to total sleep deprivation.

Recently, there has been progress in understanding mechanisms that underlie individual differences in performance-related responses to total sleep deprivation. Studies that have used fMRI have shown that individual differences in patterns of task-dependent brain activation are reproducible across multiple exposures to sleep deprivation^67^. Participants who are categorized as vulnerable to the effects of sleep deprivation on performance show reduced global brain activation at baseline when they are well rested^9–11^, as well as poorer separation of cortical networks that are functionally segregated^12^. These studies demonstrate that brain imaging markers can potentially be used to explain and predict attentional responses to total sleep deprivation. Additionally, polymorphisms in genes implicated in sleep and circadian physiology have been shown to associate with individual differences in neurobehavioral performance, either during baseline or in response to sleep deprivation^58,68–73^. It is possible that our models for predicting attentional vulnerability to total sleep deprivation would perform better if additional factors were included as predictor variables, including brain imaging markers, genotype, or physiological variables associated with attentional vulnerability to sleep deprivation^14^.

In conclusion, our analyses of laboratory data suggest that it is possible to classify participants into groups that are either more vulnerable or more resilient to the effects of total sleep deprivation on attentional lapses, using data derived from a 10-min PVT taken under rested daytime conditions. Under ideal experimental conditions, we show that the upper limit of classification accuracy is about 75%. Further improvement in predicting attentional lapses during sleep deprivation will require the discovery and implementation of new metrics. In the long term, our modeling approach and related methods can potentially be used toward developing personalized fatigue management strategies when exposure to long work hours and sleep deprivation cannot be avoided. With further development of such algorithms and testing of their predictive ability in other populations and in real-world operational environments, it might be possible to minimize the risk of attentional failures by screening for personnel resilient to the effects of sleep loss and/or deploying appropriate individualized fatigue countermeasures in those persons who are predicted to be more vulnerable to sleep deprivation.

## Supplementary information

Supplementary Information

## Data Availability

The authors will make relevant anonymized data available on reasonable request. Execution of a Materials Transfer Agreement is required if the data will be used in research supported by a for-profit company, per Partners Healthcare Institutional Review Board policy.
