# Supplementary material for: Classifying attentional vulnerability to total sleep deprivation using baseline features of Psychomotor Vigilance Test performance
Source: Sci Rep. 2019 Aug 20;9:12102. doi: 10.1038/s41598-019-48280-4 (PMC6702200; doi:10.1038/s41598-019-48280-4)
Supplement: Supplementary file 1 — Supplementary Information [file 41598_2019_48280_MOESM1_ESM.pdf]

Classifying attentional vulnerability to total sleep deprivation using baseline features of Psychomotor Vigilance Test performance

Eric Chern-Pin Chua, PhD[1,2], Jason P Sullivan, BS[3], Jeanne F Duffy, MBA, PhD[3,4], Elizabeth B Klerman, MD, PhD[3,4], Steven W Lockley, PhD[3,4], Bruce S Kristal, PhD[3,4], Charles A Czeisler, PhD, MD[3,4], Joshua J. Gooley, PhD[1,2*]

[1]Center for Cognitive Neuroscience, Duke-NUS Medical School, Singapore 169857
[2]Neuroscience and Behavioral Disorders Program, Duke-NUS Medical School, Singapore 169857
[3]Division of Sleep and Circadian Disorders, Departments of Medicine and Neurology, Brigham and Women's Hospital, Boston, MA 02115
[4]Division of Sleep Medicine, Harvard Medical School, Boston, MA 02115

***Corresponding author:**
Joshua J. Gooley, PhD
Center for Cognitive Neuroscience
Neuroscience and Behavioral Disorders Program
Duke-NUS Medical School Singapore
8 College Road Singapore 169857
Email: joshua.gooley@duke-nus.edu.sg
Phone: 65+ 6516 7430

SUPPLEMENTARY INFORMATION

Table S1

Examples of Psychomotor Vigilance Test features included in the model

**Category**	**Sub-category**	**No. of measures**	**Examples**
Speed	Mean/median Reaction Time (RT)	24	Mean log RT<br>Mean fastest 10% log RT
	Lapses	20	RT > 549 ms<br>RT > 2669 ms
	Percentile RT	24	10th percentile RT<br>90th percentile RT
Variability	Standard deviation (SD) of RT	12	SD log RT<br>SD fastest 10% log RT
	Difference of consecutive reaction times (Δ RT) exceeding a threshold	161	Δ RT > 50 ms<br>Δ RT > 800 ms
	Difference (Δ) of percentile reaction times	171	Δ 25th – 75th percentile RT<br>Δ 10th – 40th percentile RT
Time on Task	-	8	Slope of linear regression<br>Intercept of linear regression
Error	-	6	Anticipations<br>Timeouts
**TOTAL**		**426**	

Table S2

Model parameters and cost matrices at each time point for classifying vulnerability (n = 160)

<table>
<tr><th>Time point</th><th>PVT measures</th><th>Cost matrix</th></tr>
<tr><td>Wake + 4 h</td><td>(1) Median fastest 10% 1/RT
(2) lg(SD of 1/RT)
(3) lg(proportion of consecutive trials with ΔRT > 120 ms + 1)
(4) lg(Δ 5th – 40th percentile RT)
(5) lg(Δ 10th – 50th percentile RT)
(6) lg(Δ 25th – 55th percentile RT)
(7) lg(Δ 25th – 95th percentile RT)</td><td><table><tr><td></td><td></td><td colspan="3">True class j</td></tr><tr><td></td><td></td><td>R</td><td>I</td><td>V</td></tr><tr><td rowspan="3">Estimated class i</td><td>R</td><td>0</td><td>1</td><td>1</td></tr><tr><td>I</td><td>1</td><td>0</td><td>1</td></tr><tr><td>V</td><td>1</td><td>1</td><td>0</td></tr></table>C(i|j): cost of classifying as i when it is actually j</td></tr>
<tr><td>Wake + 6 h</td><td>(1) lg(Δ 25th – 60th percentile RT)
(2) lg(Δ 30th – 65th percentile RT)
(3) Fitted value at t=10min of regression line for 1/RT</td><td><table><tr><td></td><td></td><td colspan="3">True class j</td></tr><tr><td></td><td></td><td>R</td><td>I</td><td>V</td></tr><tr><td rowspan="3">Estimated class i</td><td>R</td><td>0</td><td>1</td><td>2</td></tr><tr><td>I</td><td>2</td><td>0</td><td>2</td></tr><tr><td>V</td><td>2</td><td>1</td><td>0</td></tr></table>C(i|j): cost of classifying as i when it is actually j</td></tr>
<tr><td>Wake + 8 h</td><td>(1) Median lg RT
(2) Median 1/RT
(3) lg(50th percentile RT)
(4) lg(65th percentile RT)
(5) lg(Δ 10th – 30th percentile RT)
(6) lg(Δ 10th – 35th percentile RT)
(7) lg(Δ 15th – 45th percentile RT)
(8) lg(Δ 40th – 60th percentile RT)
(9) lg y-intercept of regression line for lg RT
(10) lg y-intercept of regression line for 1/RT</td><td><table><tr><td></td><td></td><td colspan="3">True class j</td></tr><tr><td></td><td></td><td>R</td><td>I</td><td>V</td></tr><tr><td rowspan="3">Estimated class i</td><td>R</td><td>0</td><td>1</td><td>2</td></tr><tr><td>I</td><td>2</td><td>0</td><td>2</td></tr><tr><td>V</td><td>2</td><td>1</td><td>0</td></tr></table>C(i|j): cost of classifying as i when it is actually j</td></tr>
<tr><td>Wake + 10 h</td><td>(1) Median 1/RT
(2) Median slowest 10% 1/RT
(3) lg(70th percentile RT)
(4) Proportion of consecutive trials with ΔRT > 60 ms + 1
(5) lg(Δ 5th – 35th percentile RT)
(6) lg(Δ 5th – 45th percentile RT)
(7) lg(Δ 5th – 55th percentile RT)
(8) lg(Δ 10th – 55th percentile RT)
(9) lg(Δ 15th – 35th percentile RT)
(10) lg(Δ 15th – 45th percentile RT)
(11) lg(Δ 35th – 65th percentile RT)</td><td><table><tr><td></td><td></td><td colspan="3">True class j</td></tr><tr><td></td><td></td><td>R</td><td>I</td><td>V</td></tr><tr><td rowspan="3">Estimated class i</td><td>R</td><td>0</td><td>1</td><td>1</td></tr><tr><td>I</td><td>1</td><td>0</td><td>1</td></tr><tr><td>V</td><td>1</td><td>1</td><td>0</td></tr></table>C(i|j): cost of classifying as i when it is actually j</td></tr>
</table>

<table>
<tr>
<td>Wake + 12 h</td>
<td>(1) Median 1/RT
(2) lg(45th percentile RT)
(3) lg(standard deviation of middle 80% lg RT)
(4) lg(proportion of consecutive trials with ΔRT > 70 ms + 1)
(5) sqrt(number of consecutive trials with ΔRT > 130 ms)
(6) sqrt(number of consecutive trials with ΔRT > 140 ms)
(7) sqrt(number of consecutive trials with ΔRT > 210 ms)
(8) lg(Δ 5th – 40th percentile RT)
(9) lg(Δ 10th – 30th percentile RT)
(10) lg(Δ 15th – 80th percentile RT)
(11) lg(Δ 15th – 85th percentile RT)
(12) lg(Δ 20th – 55th percentile RT)
(13) lg(Δ 20th – 70th percentile RT)
(14) Fitted value at t=10min of regression line for 1/RT</td>
<td>
<table>
<tr><td colspan="2"></td><td colspan="3">True class j</td></tr>
<tr><td colspan="2"></td><td>R</td><td>I</td><td>V</td></tr>
<tr><td rowspan="3">Estimated class i</td><td>R</td><td>0</td><td>1</td><td>3</td></tr>
<tr><td>I</td><td>3</td><td>0</td><td>3</td></tr>
<tr><td>V</td><td>3</td><td>1</td><td>0</td></tr>
</table>
C(i|j): cost of classifying as i when it is actually j
</td>
</tr>
<tr>
<td>Wake + 14 h</td>
<td>(1) Mean fastest 10% 1/RT
(2) No. of RT > 549 ms
(3) lg(98th percentile RT)
(4) lg(Δ 20th – 55th percentile RT)
(5) lg(Δ 25th – 60th percentile RT)</td>
<td>
<table>
<tr><td colspan="2"></td><td colspan="3">True class j</td></tr>
<tr><td colspan="2"></td><td>R</td><td>I</td><td>V</td></tr>
<tr><td rowspan="3">Estimated class i</td><td>R</td><td>0</td><td>1</td><td>3</td></tr>
<tr><td>I</td><td>3</td><td>0</td><td>3</td></tr>
<tr><td>V</td><td>3</td><td>1</td><td>0</td></tr>
</table>
C(i|j): cost of classifying as i when it is actually j
</td>
</tr>
</table>

A 3-class linear discriminant model was developed at each baseline time point to classify participants in different vulnerability groups (vulnerable, intermediate, resilient) using features of Psychomotor Vigilance Test (PVT) performance. In the cost matrix column, R = resilient, I = intermediate, V = vulnerable. RT, reaction time; SD = standard deviation; sqrt, square root
